# Robert Stephens Fairbank

**Published:** 1910-08-06

**Authors:** 


On August 1 there died, at his residence, 13 Porchester Terrace, Hyde Park, W., Robert Stephens Fairbank, M.R.C.S. Eng., L.S.A., of 18 George Street, Hanover Square, W., and Beaumont, Bembridge, Isle of Wight. Mr. Fairbank obtained his diplomas in 1894 from King's College Medical School, and was later an Assistant Demonstrator of Anatomy and Physiology at his old school.

